# Telemedicine Use Among Older Adults During the COVID-19 Pandemic

**DOI:** 10.1093/geroni/igab046.1805

**Published:** 2021-12-17

**Authors:** Anita Szerszen, Yulia Kogan, Edith Burns

**Affiliations:** 1 Northwell Health, Staten Island University Hospital , Staten Island, New York, United States; 2 Northwell Health, New Hyde Park, New York, United States

## Abstract

Objective: Although technology adoption among older adults is improving, ethnic minorities and those with socioeconomic disadvantages may have lower utilization of telemedicine. Here, we evaluate telemedicine uptake amongst community-based older adults. Materials and Methods: Using a retrospective cohort design, we examined electronic medical records (EMR) for documentation of telemedicine use among patients > 65 years old at Geriatric practices in the New York metropolitan area from January-November 2020. Demographic details and insurance payer were captured for telemedicine visits and compared to in-person encounters. Multivariable regression was used to evaluate the association of demographic, socioeconomic factors and visit type. Results: A total of 712 patients (32.3%) engaged in 1,085 telemedicine visits. Telemedicine represented 80% and 66% of all encounters during April and May, respectively and averaged 11.8% between June and November. Use was similar across age groups, gender, race and insurance payer status between telemedicine versus in-person encounters. Patients with greater number of comorbidities were more likely to use telemedicine-. Medicaid recipients had preference for video visits. 47.5% of patients who engaged in video visits had another person/family member present during an encounter. Conclusions: Telemedicine augmented access to health care for older individuals during the peak of the COVID pandemic and continues to be utilized to improve access to care for older Americans. Given the distinct preference for video visits among patients with multiple medical conditions and those who have Medicaid, telemedicine has potential to serve as a tool to reduce enduring health care disparities beyond the pandemic.

